# Increased risk of acute pancreatitis occurrence in smokers with rs5751901 polymorphisms in GGT1 gene

**DOI:** 10.7150/ijms.38657

**Published:** 2020-01-14

**Authors:** Milena Ściskalska, Monika Ołdakowska, Grzegorz Marek, Halina Milnerowicz

**Affiliations:** 1 Department of Biomedical and Environmental Analyses, Faculty of Pharmacy, Wroclaw Medical University, Wroclaw 50-556, Poland; 2 Second Department of General and Oncological Surgery, Wroclaw Medical University, Wroclaw 50-556, Poland

**Keywords:** rs5751901, rs2236626, smoking, polymorphism, γ-glutamyltransferase, acute pancreatitis

## Abstract

**Objectives**: The study was aimed to assess γ‑glutamyltransferase (GGT) activity and concentration as a marker of oxidative stress induced by exposure to tobacco smoke in acute pancreatitis (AP) course. Examination of the relationship between GGT activity/concentration and single-nucleotide polymorphism (SNP rs5751901 and rs2236626) in GGT1 gene was performed.

**Subjects and methods**: We examined SNPs in 38 AP patients and 51 healthy subjects by PCR-RFLP methods. GGT concentration in blood was measured with the use of the ELISA method; GGT activity and GSH concentration were measured by the Szasz and Patterson methods, respectively.

**Results**: In the non-AP smokers group with TC genotype for SNPrs5751901 an increased blood GGT activity compared to smokers with CC genotypes was shown. In the course of AP was observed an elevated GGT activity and the value of GGT activity/GGT concentration ratio in smokers compared to non-smokers, in AP patients with TC genotypes and CC genotypes, respectively, for both SNP: rs5751901 and rs2236626. In the group of smoking AP patients with the CC and TC genotypes in rs5751901 locus and CC and TT genotypes in rs2236626 locus a decreases in GSH concentration during hospitalization were noted.

**Conclusions**: SNP rs5751901 and rs2236626 cause changes in GGT activity. Smoking in the AP course contributes to increased GGT activity and excessive GSH use up in patients with TC and CC genotypes for both SNPs. Exposure to smoke xenobiotics enhances (3-fold) the risk of AP occurrence in individuals with TC genotypes for SNP rs5751901.

## Introduction

Acute pancreatitis (AP) is an irreversible inflammatory process of the pancreas. About 5% cases of AP are chronic; more than 20% of them have recurrent inflammation 1. Disease results in a clinical spectrum of diseases ranging from mild and self-limiting to severe, progressing disease associated with high risk of mortality 2. Major risk factors for acute pancreatitis are gallstones (45% of cases) and alcohol abuse (30% of cases) 3,4. It has been shown that oxidative stress plays an important role in pathogenesis of acute pancreatitis 5. The potential of oxidative stress towards poor antioxidant status of pancreas causes damage of pancreatic cells 2.

For years our research has been focused on pro/antioxidant status in pancreatic diseases 6-9. It was also observed that some antioxidants may be useful for differentiation of the inflammatory processes of the pancreas through their involvement in neutralization of oxidative stress 6. Pro/antioxidative balance in pancreatic diseases seems to play a significant role in explaining the dynamics of inflammatory changes.

Our previous studies, conducted in the population of patients with pancreatitis, showed an important role of oxidative stress induced by cigarette smoke in the progression of inflammation 9,10. One of many enzymes involved in xenobiotics detoxification is γ-glutamyltransferse (GGT) (EC 2.3.2.2) - a membrane-bound enzyme occurring in different tissues, but most abundantly in kidneys, liver and pancreas. GGT is anchored to the cell surface by small N‑terminal transmembrane domain 11. In humans, the active enzyme is coded by the GGT1 gene localized on chromosome 22 (22q11), synthesized as a catalytically inactive single polypeptide, and is post-translationally processed to form heavy (H) and light (L) protein chains 11,12.

Removal of xenobiotics from cells occurs via their conjugation with glutathione (GSH) which is catalyzed by glutathione S-transferase. Then, GGT catalyzes the hydrolysis reaction of γ-glutamyl moiety from glutathione S-conjugates. Xenobiotics detoxification mediated by GGT leads to a decrease in GSH concentration 3,14. On the other hand, GGT is also key to glutathione homeostasis 11. Its main physiological function is to make cysteine available for regeneration of intracellular GSH, and hence to protect the cell against oxidative stress. GGT takes part in transport of amino acids through the cell membrane, which results in formation of cysteinylglycine that has a strong ability to reduce transition metals and generate free radicals 15. Therefore, GGT is involved in generation of free radicals and it can be recognized as a marker of oxidative stress 13,15.

The subject of this study is to examine the influence of exposure to tobacco smoke and genetic factors influencing the activity and concentration of GGT, the relation of GGT level with inflammatory state parameters and its association with the risk of acute pancreatitis occurrence. Assessment of the effects of exposure to smoke and identification of genetic polymorphisms influencing blood GGT should contribute to better understanding of the organism response to oxidative stress and the causes of inter-individual differences in the course of acute pancreatitis. The aim of this study was to assess the influence of SNP in the GGT1 gene (rs5751901 and rs2236626) on the GGT activity in blood of non-smoking and smoking patients with AP and healthy subjects. The dynamics of GGT activity/concentration changes in the course of AP were also assessed with respect to individual genotype for SNP rs5751901 and rs2236626. In the study the effect of SNPs in the GGT1 gene on GSH use up as an important small molecular antioxidant present in all tissues of the organism was assessed.

## Materials and Methods

### Subjects

The study group consisted of 38 patients with acute pancreatitis (AP) (15 non-smokers and 23 smokers) hospitalized in the Second Department of General and Oncological Surgery, Wroclaw Medical University, and 51 healthy volunteers (26 non-smokers and 25 smokers) classified as the control group. The subjects were enrolled in the study between January 2014 and December 2017. All the procedures were performed in compliance with the relevant laws and institutional guidelines. The study protocol was approved by Local Bioethics Committee of Wroclaw University of Medicine (No: KB-592/2013 and KB-529/2018). The study conforms to recognized standards.

The patients were included in the AP group based on clinical symptoms (acute onset of a persistent, severe, epigastric pain with tenderness on palpation during physical examination), personal interview and clinical methods used in diagnosis of pancreatitis - laboratory tests (three-fold elevation of serum lipase or amylase, or elevation above the upper limit of normal) and characteristic findings of acute pancreatitis on imaging (contrast-enhanced computed tomography (CT), magnetic resonance imaging (MRI) or transabdominal ultrasonography). In patients with characteristic abdominal pain and three-fold elevation of serum lipase or amylase, or elevation above the upper limit of normal, no imaging was required to establish the diagnosis of acute pancreatitis. In patients with abdominal pain that was not characteristic for acute pancreatitis, or serum amylase or lipase levels that were less than three times the upper limit of normal, or in whom the diagnosis was uncertain, we performed abdominal imaging with a contrast-enhanced abdominal CT scan to establish the diagnosis of acute pancreatitis and to exclude other causes of acute abdominal pain. In patients with severe contrast allergy or renal failure we performed abdominal MRI without gadolinium. During hospitalization intensive intravenous fluid treatment was administered, based on the calculated individual needs, taking into account the general status and the comorbidities, and adjusted by monitored vital signs, blood morphology parameters and daily urine output (on average about 4 liters of crystalloid solutions). In the cases of severe and moderate acute pancreatitis fluids were applied under strict control of RR/HR, hematocrit, hourly diuresis, and then were modified relatively to the preliminary dose of intravenous fluids (5-10 mL/kg/h) and the degree of hydration, with attention to any signs of fluid overload being paid. Patients were treated with appropriate dosage of analgesics. There was no empiric antibiotic therapy routinely applied. The patients were initially on npo (null per os) regimen, with prompt oral fluids initiation and early introduction of low fat diet monitored by symptoms resolution and laboratory test improvement. The exclusion criteria consisted of accompanying diseases, such as cancer, diabetes, liver diseases, arthritis and other chronic inflammatory diseases. None of the AP patients qualified to the study group died within seven days of hospitalization.

**Figure 1:**
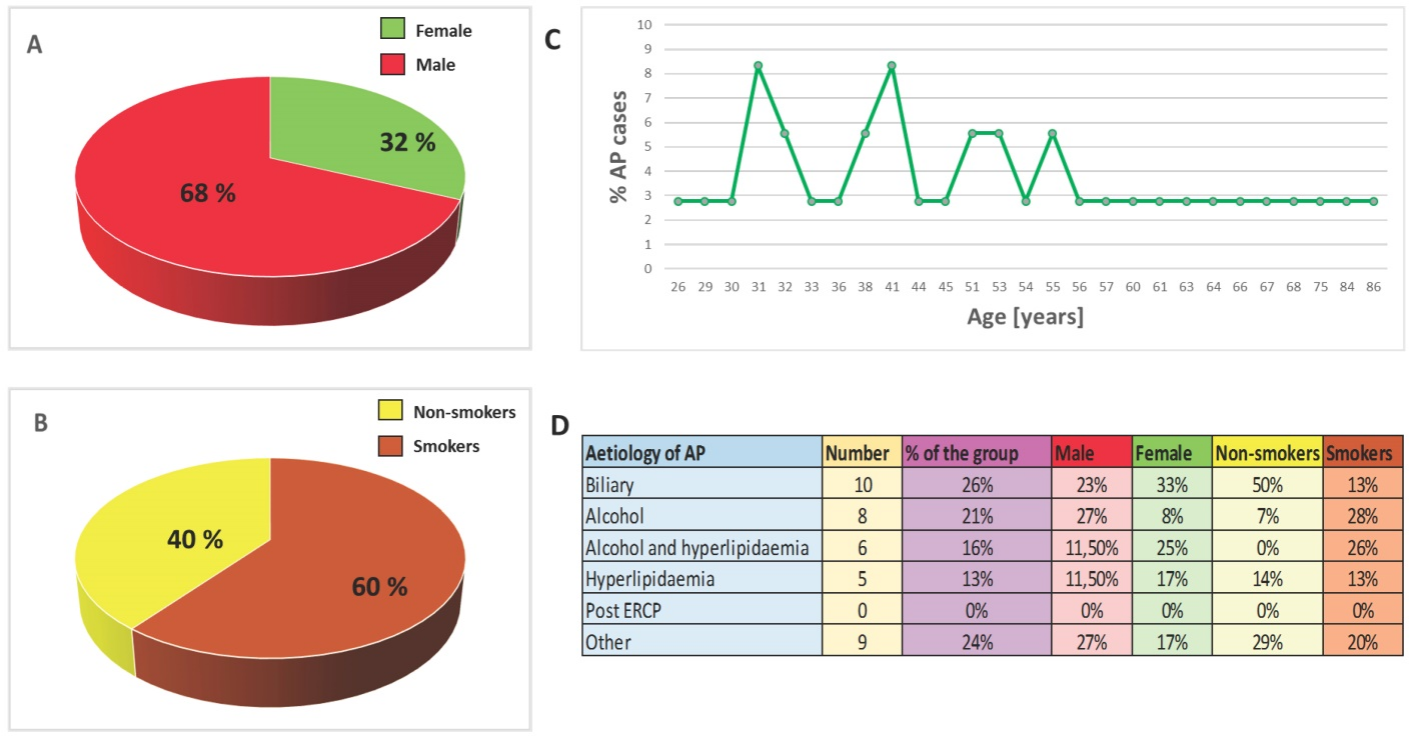
** Epidemiology and aetiology. A.** Sex distribution of AP cases. **B.** Smoking distribution of AP cases. **C**. Age distribution of AP cases. **D.** Aetiology of AP.

Healthy volunteers were enrolled as the control group based on the survey and testing performed by primary care physicians. Individuals with diagnosed disease and drug abusers were excluded from the control group. All hospitalized patients and healthy volunteers had been informed about the aim of the study and gave their written consent. Personal interview about lifestyle was carried out: participants answered questions about their health and nutritional habits, any use of dietary supplements/medications and smoking history (duration of smoking, the number of cigarettes smoked per day, smoking cessation, the occurrence of smoking-related diseases, passive exposure to cigarette smoke). Basic anthropometrical assessment was also performed. The patients were categorized into groups of smokers and non-smokers on the basis of their smoking history, and verified by determination of serum cotinine - a metabolite of nicotine - concentrations. Samples of patients and control groups were divided into two subgroups: smokers (cotinine concentration >15 ng/mL) and non-smokers (cotinine concentration <15 ng/mL); Table 1 and Figure 1 present the characteristic of the study population.

### Sample preparation

The examinations were conducted in serum, plasma and erythrocyte lysate derived from patients with AP and the control group. Blood samples were collected from the AP patients upon their admission to the hospital and on the 3^rd^ and the 7^th^ day of hospitalization. In the control group venous blood was collected in the morning, after 12 hours of fasting. The serum was obtained according to the standard procedure by drawing venous blood into disposable trace element-free tubes (Ref. No.: 368815, Becton Dickinson, Germany) with serum clotting activator, left at 25°C to complete thrombosis and centrifuged (1200g/20 min). In order to obtain samples for analysis of GSH, 1050 µl of distilled water was added to 150 µl of whole blood drawn into tubes containing heparin (Ref. No.: 368886, Becton Dickinson, Germany), then mixed and incubated for 10 min. Next, 300 µl of 25% metaphosphoric acid (Ref. No.: 253-433-4, Sigma-Aldrich, Germany) was added, mixed and incubated for 10 min and centrifuged (3,000g/10 min). The obtained samples were portioned and stored in sealed tubes (Ref. No.: 0030102.002, Eppendorf, Germany) at -80 °C until analysis.

### Methods

Cotinine was measured using the commercial Cotinine ELISA test (Ref. No.: EIA-3242, DRG International, Inc., USA). It provides qualitative screening results for cotinine in human serum at a cut-off concentration of 15 ng/mL.

High-sensitivity CRP (hsCRP) concentration was determined in the serum by turbidimetric method using C-reactive protein hs test (Ref. No.: 31927, Biosystems, Spain).

Gamma-glutamyl transferase (GGT) activity (EC 2.3.2.2) in the serum was measured by the method of Szasz 16 using a reagent for quantitative determination of GGT activity (Ref. No.: 1-228-0060, BioMaxima, Poland). GGT is the enzyme which catalyzes the transfer of the γ-glutamyl group of L-γ-glutamyl-3-carboxy-4-nitroanilide to glycyl-glycine, and produces L-γ-glutamyl-glycylglycine and 5-amino-2-nitrobenzoate. The rate of formation of 5-amino-2-nitrobenzoate is directly proportional to GGT activity at λ=405 nm. One unit of GGT is the amount of the enzyme which catalyzes the transfer of 1.0 µmol of the γ-glutamyl group from L-γ-glutamyl-3-carboxy-4-nitroanilide to glycylglycine per 1 min at 37°C. Values of GGT activities were shown in U/L.

Concentration of GGT in plasma was measured by the ELISA method with commercial test (Ref. No.: SEB375Hu, Cloud-Clone Corp., USA).

GSH concentration was measured by the method of Patterson 17. The method is based on the reaction of GSH with alloxan (Ref. No.: 23 437-0, Sigma-Aldrich, Germany) and formation of a complex with an absorbance maximum at λ=305 nm as described earlier 18.

Cd concentration was determined in erythrocyte lysate by graphite furnace atomic absorption spectrometry as described in 10.

The concentrations of oxidative stress markers (oxidized low density lipoproteins (oxLDL) in serum, malonyldialdehyde (MDA) in plasma and advanced oxidation protein products - AOPP in plasma) were determined according to the method described in 10,19.

#### Genotyping analyses

Genomic DNA was extracted from buffy coat samples (isolated from whole blood which was drawn into tubes containing disodium EDTA, Ref. No.: 367864, Becton Dickinson, Germany) using commercial kit (Syngen Blood/Cell DNA Mini Kit, Ref. No.: SY221012, Syngen, Biotech, Poland) and following the manufacturer's manual. The extracted DNA was measured using µDrop Plate (Cat. No.: N12391, Thermo Scientific) at λ=260 nm. Analysis of GGT1 polymorphisms was done by polymerase chain reaction (PCR) and restriction fragment length polymorphism analysis (PCR - RFLP). The cases and controls were genotyped for two SNPs in GGT1: rs5751901 and rs2236626. The data regarding the type of polymorphism and localization of the studied polymorphic loci were presented in Table 2. The final volume of PCR reaction mixture was 20 µl and consisted 100 pmol of each forward and reverse primer, 2 µl extracted DNA, 12,8 µl molecular clean water and 4 µl Gold Hot Start PCR Mix (Ref. No.: SY550231, Syngen) containing Taq polymerase, reaction buffer, MgCl_2_, 10x modifier GC, dNTP. The initial denaturation was accomplished at 95°C for 15 min followed by 35 cycles of 40 s at 94°C, 35 s at 62°C, 40 s at 72°C with final elongation step at 72°C for 15 min. The PCR products were digested with 10 U/µL restriction enzyme RsaI (Ref. No.: ER1121, Thermo Fisher Scientific) in buffer Tango respectively at 37˚C for 16 hours. The digested fragments were visualized in 1.5% agarose gel (Ref. No.: SY521011) with Green DNA Gel Stain (Ref. No.: SY521032, Syngen).

### Statistical analysis

The data were presented as median values. The normality of the variables was tested using the Shapiro-Wilk W test. Differences between the groups were tested using the Student's t‑test (normal distribution) or nonparametric U Mann-Whitney test (lack of normal distribution) for continuous variables, and χ^2^tests for categorical variables. To assess the dynamics of changes of the examined parameters during hospitalization of the patients with AP, the Wilcoxon's test was used. In order to verify correlations between the examined parameters, the multiple linear regression models were performed. In all instances, p<0.05 was considered statistically significant. Statistical calculations were done using the Statistica Software Package, version 13.1 (Polish version: StatSoft, Krakow, Poland).

## Results

### GGT activity in the non-smoking and smoking AP patients and in the healthy subjects

**Figure 2:**
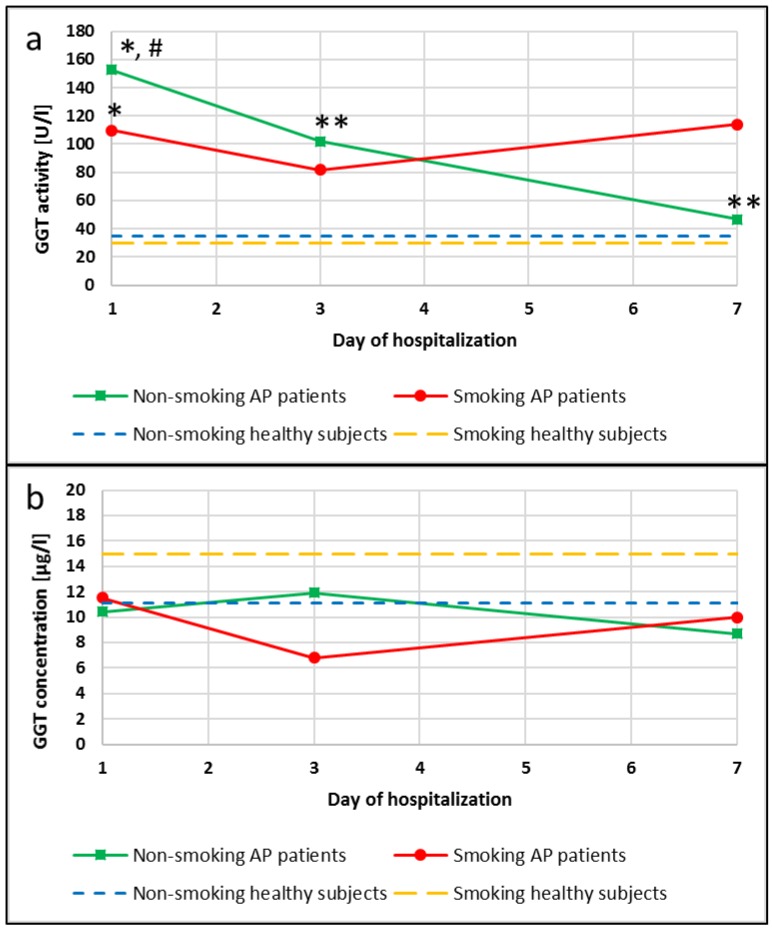
** GGT activity (a)** and concentration **(b)** in the blood of healthy subjects and the patients with AP in the 1^st^, 3^rd^ and 7^th^ day of hospitalization. #p<0.05 compared to smokers *p<0.05 compared healthy subjects **p<0.05 compared to the 1^st^ day of hospitalization.

An increase in GGT activities compared to appropriate groups of healthy subjects was observed in the blood of patients with AP. A 4-fold increase in the activity of this enzyme occurred in the group of patients with AP who were non-smokers (p<0.0001), and an increase that was bigger than 4-fold occurred in smoking patients with AP (p<0.0001), when compared to non-smokers and smokers in the control group, respectively (Figure 2a). Additionally, changes in the GGT activity during hospitalization of the AP patients were observed. The activity of this enzyme was 1.5-fold and 3.5-fold lower on the 3^rd^ and the 7^th^ day of hospitalization compared to the 1^st^ day of hospitalization in the AP group of the non-smoking patients (p=0.0453 and p=0.0042, respectively). In the smoking AP patients these changes were not observed (Figure 2a).

In the group of non-smokers no differences between GGT concentration in the healthy subjects and the AP patients were noticed. However, it was observed that GGT concentrations were decreased in the AP smokers compared to smokers in the control, but these differences was not statistically significant (Figure 2b).

### Results of genotyping

**Figure 3:**
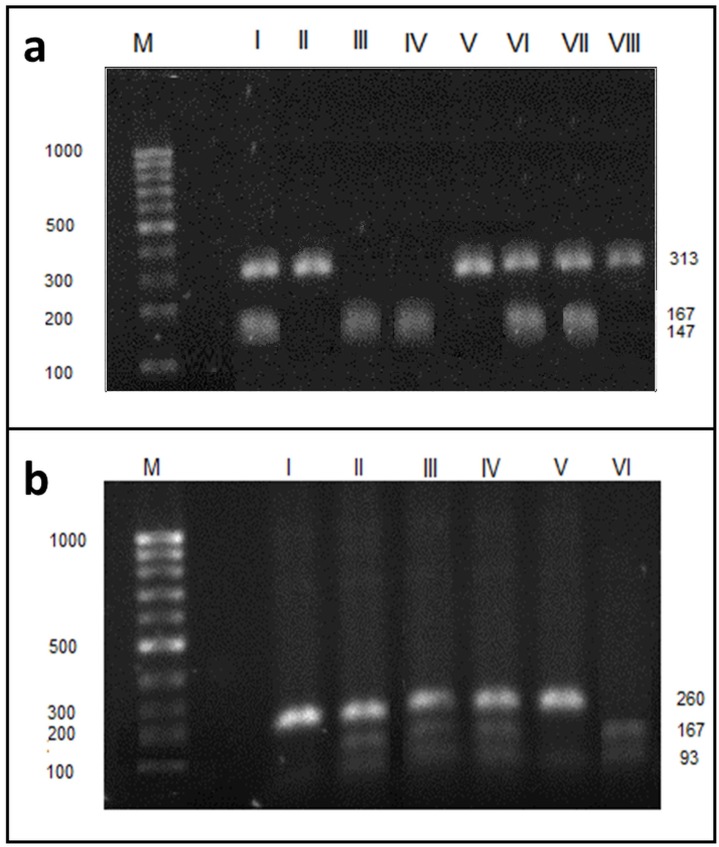
Electrophoresis pattern of GGT1 rs2236626 (a) and rs5751901 (b) polymorphism using PCR—RFLP and RsaI restriction enzyme. M - marker ladder (100-1000 *bp*); **a** - II, V, VIII - undigested PCR products (313 *bp* fragment), I, VI, VII - TC (313, 167 and 147 *bp* fragments), III, IV - CC (167 and 147 *bp* fragments). **b -** I, V - undigested PCR products (260 *bp* fragment), II-IV- TC (260, 167 and 93 *bp* fragments), VI - CC (167 and 93 *bp* fragments). Numbers are in base pair (*bp*).

We analyzed the GGT activity with respect to single nucleotide polymorphism (SNP). After genomic DNA of the samples was amplified by PCR, the target 260-bp nucleotide sequences (for SNP rs5751901) and 313-bp (for SNP rs2236626) could be seen in all samples. The identified genotypes were labelled according to the presence or absence of the enzyme restriction site for SNP rs2236626. Thus, the TT genotype is homozygous in the absence of the site (band at 313-bp), the TC genotype is heterozygous in the presence and absence of the site (band at 147-, 166- and 313-bp) and the CC genotype is homozygous in the presence of the site (band at 147- and 166-bp - Figure 3a). For SNP rs5751901, the TT genotype is homozygous in the absence of the site (band at 260-bp), the TC genotype is heterozygous in the presence and absence of the site (band at 93-, 167- and 260-bp) and the CC genotype is homozygous in the presence of the site (band at 93-, 167-bp) (Figure 3b). The frequency of occurrence of the genotypes for SNPs in the study population was presented in Table 3.

### Genotypes and dynamic of the changes in the activity and concentration of GGT in the non-smoking and smoking AP patients during hospitalization

**Figure 4:**
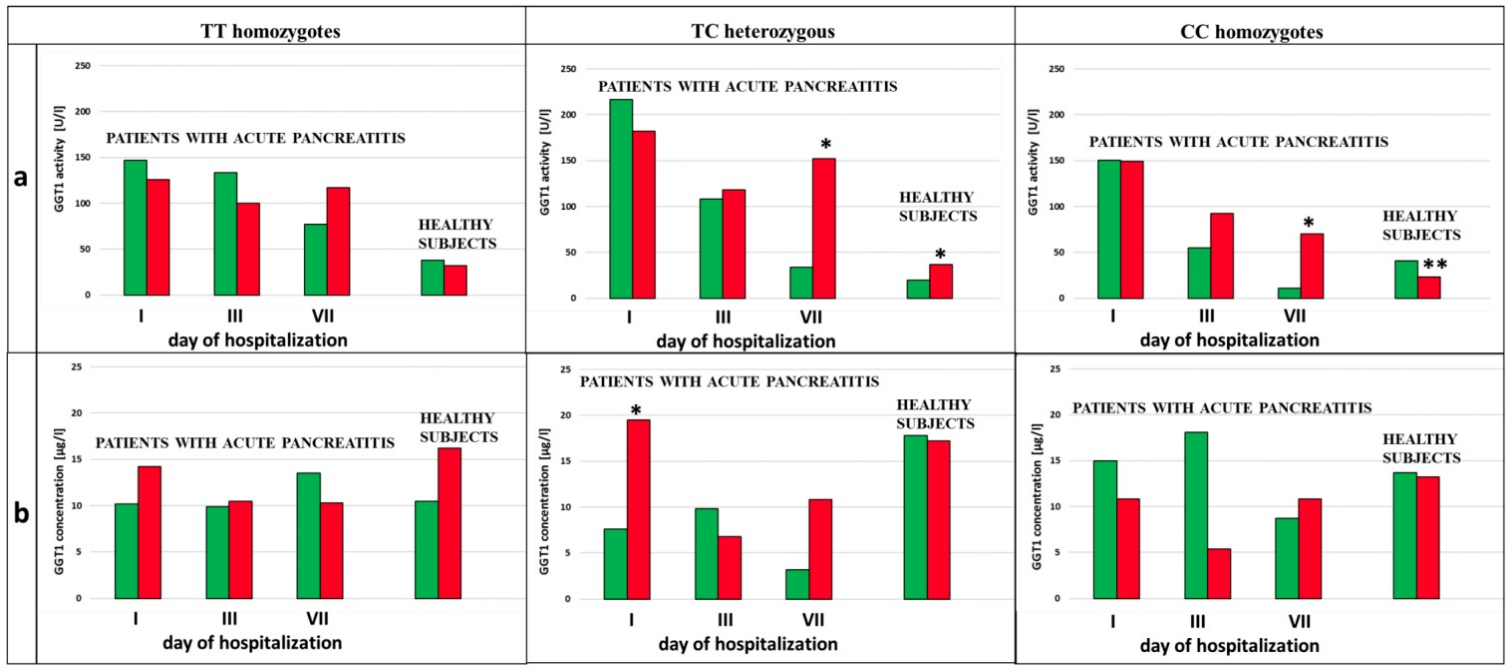
GGT activity **(a)** and concentration **(b)** in terms of SNP rs5751901 in non-smoking and smoking patients with AP and healthy subjects. 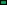 Non-smokers 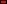 Smokers * p<0.005 compared to non-smokers ** - p<0.005 compared to smokers with TC genotypes.

We analyzed the influence of exposure to tobacco smoke on the GGT activity in the subjects with each type of genotype for the examined SNPs (rs5751901 and rs2236626). In the control subjects an increased GGT activity was observed in smokers compared to non-smokers with the TC genotype for SNP rs5751901 (p=0,0321). This activity remained significantly increased compared to the activity of this enzyme in smokers with the CC genotype for rs5751901 (p=0,0496) (Figure 4a). Similar changes in the GGT activity were also shown in the AP patients. The GGT activity in the smoking AP patients with the TC and CC genotypes for SNP rs5751901 increased by more than 5 times compared to the non-smokers in this group on the 7^th^ day of hospitalization (p=0,0455 and p=0.0177, respectively) (Figure 4a). Additionally, higher GGT concentration in the smoking AP patients with the TC genotypes for SNP rs5751901 was observed compared to the non-smokers on the 1^st^ day (p<0.0001) (Figure 4b).

**Figure 5:**
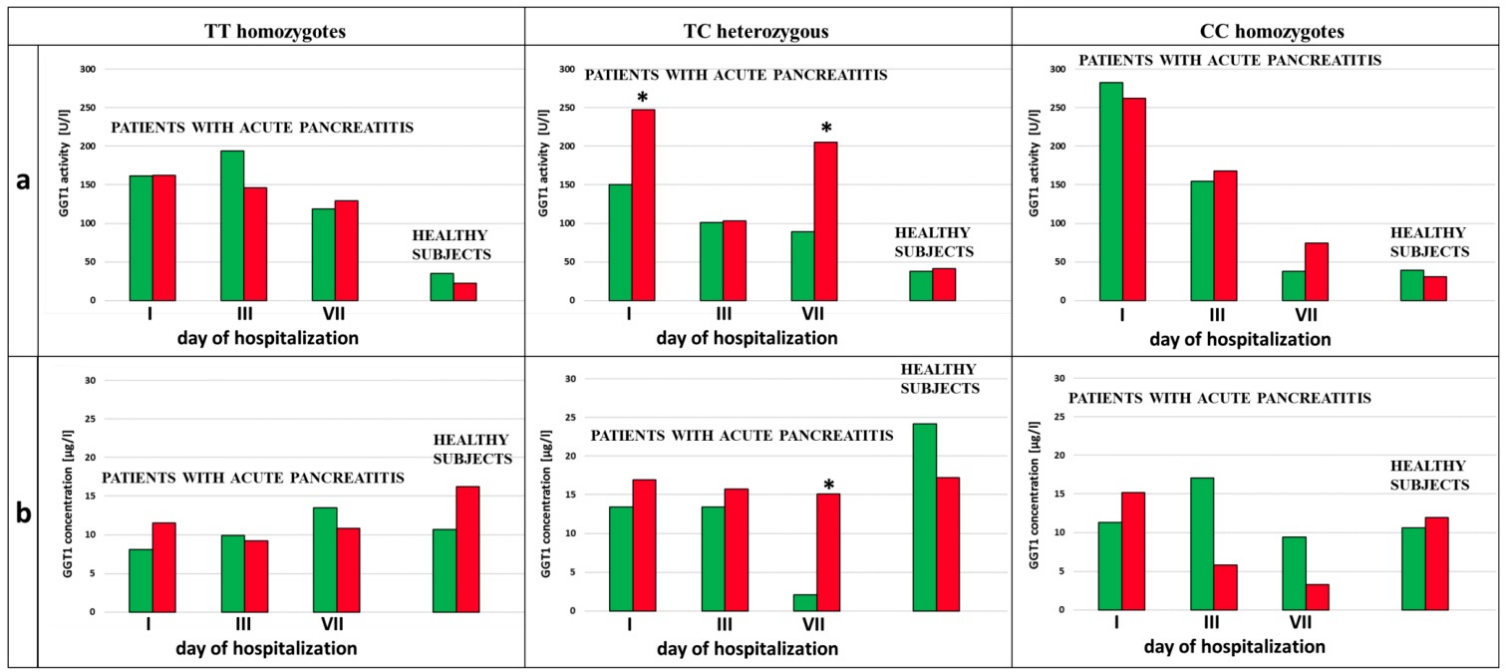
GGT activity (a) and concentration (b) in terms of SNP rs2236626 in non-smoking and smoking patients with AP and healthy subjects. 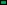 Non-smokers 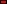 Smokers * p<0.005 compared to non-smokers.

No differences in the GGT activity between the smokers and the non-smokers with the TT, TC and CC genotypes for SNP rs2236626 in the control group were noticed (Figure 5a). However, in the group of AP patients with TC genotypes for SNP rs2236626, an elevated GGT activity in the smokers was observed compared to the non-smokers on the 1^st^ (p=0,0022) and the 7^th^ (p=0,0161) day of hospitalization (Figure 5a). Additionally, in this group it was observed that an increase in the GGT activity on the 7^th^ day of hospitalization was accompanied by elevated GGT concentration in the smokers compared to the non-smokers (p<0.0001) (Figure 5b).

**Figure 6:**
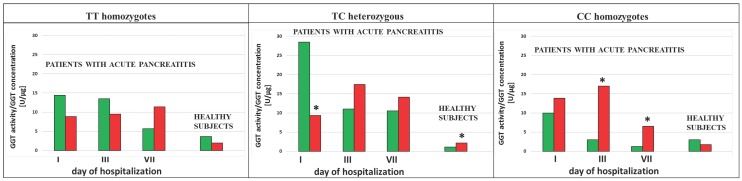
The ratio of GGT activity/GGT concentration in terms of SNP rs5751901 in non-smoking and smoking AP patients and healthy subjects. 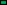 Non-smokers 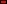 Smokers * p<0.05 compared to non-smokers.

**Figure 7:**
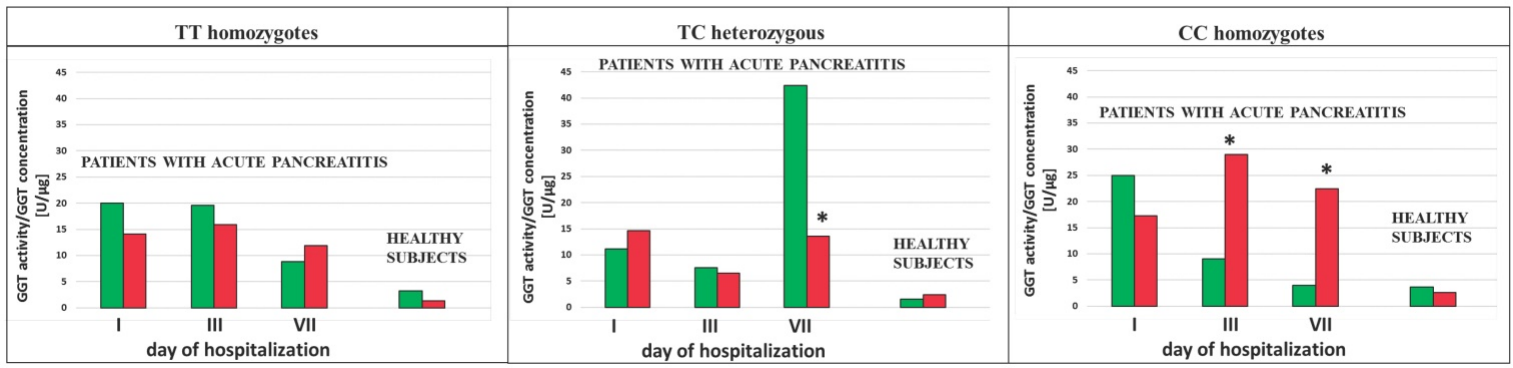
The ratio of GGT activity/GGT concentration in terms of SNP rs2236626 in non-smoking and smoking AP patients and healthy subjects. 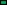 Non-smokers 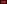 Smokers * p<0.05 compared to non-smokers.

The ratio of the GGT activity/GGT concentration was calculated in the study population. This ratio showed decreased value in AP patients in the group of smokers as compared to the group of non-smokers with the TC genotypes (p<0.0001 for the 1^st^ and the 3^rd^ day of hospitalization and for SNP rs5751901 and rs2236626, respectively) (Figure 6 and 7). However, in the smoking healthy subjects with the TC genotypes for SNP rs5751901 an increase in the ratio of GGT activity/GGT concentration compared to the group of non-smokers was observed (Figure 6). A similar relation between the smokers and the non-smokers was seen in the blood of AP patients in the CC genotypes for SNP rs5751901 and rs2236626 on the 3^rd^ (p=0.0301 and p<0.0001) and on the 7^th^ day of hospitalization (p=0.0322 and p<0.0001) (Figure 6 and 7).

### Genotypes and changes in the dynamics of GSH concentration in the non-smoking and smoking AP patients during hospitalization

Increased GSH concentration in the smoking AP patients compared to the non-smokers with the CC genotypes for SNP rs5751901 (p=0,0003, p=0,0126 and p<0,0001 on the 1^st^, the 3^rd^ and the 7^th^ day of hospitalization) and the TC genotypes was observed (p=0,0094 for the 3^rd^ day of hospitalization) (Table 4). Additionally, the lowest GSH concentrations were observed in the non-smoking AP patients with the CC genotypes for SNP rs5751901 which were statistically significant as compared to TT homozygous on the 3^rd^ (p=0,0355) and the 7^th^ day of hospitalization (p<0,0001) and TC heterozygous on the 7^th^ day (p<0,0001). However, in the group of the smoking AP patients with the CC and TC genotypes a decrease in GSH concentration during hospitalization was noted (p=0,0324 and p=0,0381 compared in the 1^st^ and 7^th^ day) (Table 4).

Increased GSH concentration was observed in the smoking AP patients compared to the non-smokers with the CC genotypes for SNP rs2236626 (p<0,0001 on the 1^st^ and the 7^th^ day of hospitalization) and the TC genotypes (p=0,0221 and p=0,0068 on the 1^st^ and the 3^rd^day of hospitalization) (Table 4). GSH concentration in the smokers was decreasing during hospitalization of the AP patients with the TT genotypes (p=0,0289 on the 1^st^ and the 7^th^ day) and the CC genotypes for this SNP (p=0,0313 on the 1^st^ and the 3^rd^ day). However, in the non-smoking group of AP patients with the TC genotypes an increase in GSH concentration on the 7^th^ day, compared to the 3^rd^ day of hospitalization, was seen (p=0,0055). Additionally, in the non-smoking group of AP patients with the TT genotypes for SNP rs2236626 the highest GSH concentration was observe which was statistically significant comparing to the CC and TC genotypes (respectively: p=0,0314 and p=0,0388 on the 1^st^ day of hospitalization) (Table 4).

### The results for the odds ratio analysis and the correlation coefficients

In this study we examined the association between AP occurrence and exposure to tobacco smoke in subjects with SNP rs5751901 and rs2236626. In the smokers group with the TC genotype for SNP rs5751901 the risk of AP recurrence was elevated by more than three times (OD=3,1364, p=0.0471). In the case of the TT and CC genotypes this association was not statistically significant (OD=0,5625, p=0,2936 and OD=1,3125, p=0,7589, respectively). The association between AP occurrence and smoking in the subjects with the TT, CC and TC genotypes for SNP rs2236626 was not observed (OD=2,0125, p=0,2761; OD=1,0385, p=0,9702 and OD=1,9000, p=0,3884 respectively).

In the smokers group with AP, statistically significant correlations in terms of genotype for SNP rs5751901 and rs2236626 were noticed as presented in Table 5.

## Discussion

Recent studies underline the significance of GGT in the induction and progression of inflammation and oxidative stress 20-22. It is in agreement with the findings of our study in which strong positive correlations of inflammatory state parameters were shown in the patients with AP. The correlation between the GGT activity and the oxidative stress markers indicates that the course of acute pancreatitis is accompanied by intensive oxidative stress and confirms the possible use of GGT as an oxidative stress marker. In other studies it was shown that oxidative stress can contribute to cell death by ischemic stroke and that it can be considered to be an important regulator of pathogenesis of acute pancreatitis 23,24.

GGT can be considered as a marker of tissue damage in the liver and the pancreas induced by exposure to xenobiotics 13,25,26. In our study it was found that exposure to smoke xenobiotics significantly influenced the dynamics of changes in the GGT activity during hospitalization. The GGT activity was normalizing quicker in the non-smokers with AP compared to the smokers. The elevated GGT activity of the smokers can reflect increased exposure to xenobiotics which are metabolized in the liver through glutathionylation and then detoxified with the participation of GGT 14. In our study there was a statistically significant increase in blood Cd levels in the smokers compared to the non-smokers. Also, there was a strong correlation between the GGT activity and the Cd levels or cotinine concentrations observed, which confirms toxic effects of heavy metals present in tobacco smoke being their main source in blood. Moreover, exposure to tobacco smoke is an important factor of intensifying oxidative stress in the course of AP. In this context, elevation of the GGT activity in AP patients may reflect exposure to oxidative stress related to both smoke xenobiotics and the course of the disease. It can result in pancreatic inflammation-related cytokine release intensifying pre-existing inflammation. While analyzing the GGT activity as a marker of oxidative stress we need to remember that even though we observed its increase in the smokers as compared to the non-smokers, the much stronger cause of it lies in the inflammatory course of AP itself.

The increase of the GGT activity in our study, however, did not correspond to the increase of GGT concentration. To explain the causes of the increased GGT activity in AP patients compared to healthy subjects, and its different dynamics between the smokers and the non-smokers, assessment of single-nucleotide polymorphisms (SNPs) was performed in the study population. Significant effect of SNPs in the GGT1 gene (rs5751901 and rs2236626) on the GGT activity in blood was confirmed, which was also found in earlier studies 27,28. Our results demonstrated highest GGT activity in the AP patients with the TT and TC genotypes for SNPrs5751901 and the TC and CC genotypes for SNP rs2236626 on the first day of hospitalization (data shown in Supplementary Materials). The influence of SNPs on the GGT activity was also shown when the study population was divided into smokers and non-smokers. The smokers in the control (non-AP) group with the TC genotypes for SNPrs5751901 had an increase in the GGT activity compared to subjects with the CC genotypes. It can indicate that occurrence of single-nucleotide polymorphism can also have influence on steric conformation of enzyme molecule 29. Both SNPs examined in this study are localized in gene sequence coding a large subunit of the GGT molecule which is wrapped around the small subunit 30. However, it is known that substrate binding to the GGT molecule can cause a change of conformation for the side chain of the catalytic residue, Thr-381, localized in the cleft of small subunit. SNPs occurrence in the large subunit could result in uncorrected change of GGT conformation during substrate binding. It may cause changes in availability of thiol group in catalytic center of GGT, changes of its affinity for the enzyme substrate and subsequently differences in transpeptidase GGT activity (like the observed in this study).

Our results have shown that smoking contributes to increase in the GGT activity in AP patients with the TC genotypes for both SNPs (rs5751901 and rs2236626). It could reflect elevated metabolism of smoke xenobiotics via the above-mentioned mechanism, and confirm its toxic effect on cells. In the smokers with the TC genotypes, increase in the GGT activity could be a result of slightly increased GGT concentration. SNPs can make changes in amino acids encoding, which may have an effect on promoter activity, gene expression and stability of mRNA or its subcellular localization, influencing GGT concentration 31. SNPs can be also related to up-regulation GGT mRNA at transcription level under oxidative stress conditions. There is evidence that the GGT gene expression in animals and humans is controlled by redox mechanisms and signal pathways activated in response to oxidative stress 15,32,33. Zhang et al. 33 described that this pathway includes, among others, activation of protein kinase C, mitogen-activated protein kinase and activation of protein 1-binding element which are also induced by smoke xenobiotics 34. The correlations between GGT and oxidative stress markers showed in the groups of smokers could support this thesis. It can indicate the role of GGT as a marker of oxidative stress induced by tobacco smoke xenobiotics. In the AP patients with the TC genotypes for both tested SNPs it was also observed that recurrent course of AP occurred only in smokers (data not shown). This group of AP patients showed the weakest tendency to normalize the GGT activity during hospitalization. Additionally, tobacco smoking in the subjects with the TC genotypes for SNP rs5751901 elevated the risk of AP occurrence by more than three times. It can be associated with pro-inflammatory effect of smoke xenobiotics via free radicals pathway, which was reported in other papers 34,35.

In the smoking group of AP patients with the CC genotypes for SNPrs5751901 and rs2236626, however, a disproportional increase in the GGT activity in comparison to the concentration of this protein was observed, which was reflected by the significantly increased ratio of the GGT activity/GGT concentration. This finding can suggest that smoking in the CC genotype group may have influence on protein incorporation into the cell membrane. Subsequently, the occurrence of this polymorphism in the heavy chain of the GGT molecule can weaken its anchoring to the cell membrane, simultaneously sensitizing it to oxidative stress damage. This was reflected in the study conducted by Koregol et al. 36 in which GGT served as biomarker for cellular damage caused by oxidative stress. One of the possible causes of this weakening can be the change of availability of sialic acid residues, found only in the heavy subunit and responsible for bounding the GGT molecule to the cell membrane 37. Thus, our results can indicate that the exposure to smoke xenobiotics in the CC genotypes for examined SNPs may influence the intensity of oxidative stress and change the tertiary and quaternary protein structure of the GGT molecule. It can results in release of the enzyme from the cell membrane, which influences its activity. Therefore, disproportional increase in the GGT activity in comparison to GGT concentration may indicate impairment of GGT functions as a membrane protein.

Our results confirmed that tobacco smoking caused continuation of GSH reserves use up by GGT in spite of treatment. It was shown in gradual decrease of GSH concentration during hospitalization of the smoking AP patients with the CC and TC genotypes for SNP rs5751901 in whom increased GGT activity, compared to the non-smokers, was shown. The increases in the GGT activity in the blood of the smokers can happen in response to oxidative stress facilitating transport of more GSH precursors into the cells. This highlights the role of GGT in the maintenance of intracellular antioxidant defenses through mediation of extracellular GSH transport into the cells 38. The intensified GSH usage in this group of AP patients can impair neutralization of the free radicals and can aggravate the course of the disease. However, an increase in GSH concentration in the course of AP in the non-smokers with the TC genotypes for SNP rs2236626 can indicate normalization of GSH reserves in response to silencing inflammation and oxidative stress associated with it.

In this study, impairment of GSH hemostasis in the course of AP was shown. Significantly decreased GSH concentrations in the blood of AP patients compared to the healthy subjects (median 288.4 µg/mL according 39) can be a result of changed protein metabolism in acute phase of inflammation 40. In other studies it was shown that the liver plays the central role in the inter-organ GSH homeostasis 41. The increase in GSH concentrations in the smokers compared to the non-smokers with AP observed in this study can be recognized as an adaptive response to cigarette smoke exposure. It is known that smoke xenobiotics can contribute to inhibition of endothelial nitric oxide (NO) synthase (eNOS) and reduce basal endogenous NO synthesis in blood vessels of smokers 42. Other researches showed that nitric oxide and GSH are involved in induction of hepatic synthesis of stimulants of pancreatic secretion 43. In the case of impairment of pancreatic secretion caused by exposure to tobacco smoke xenobiotics in the course of AP, an increase in GSH concentration can be recognized as a compensatory mechanism which is responsible for maintenance of pancreatic function in intensified oxidative stress conditions. In spite of increased GSH concentration in the smokers with AP compared to the non-smokers, a trend of decrease of its concentration during hospitalization was observed.

In summary, in this study it was confirmed that single nucleotide polymorphism in the GGT1 gene encoding the large subunit of GGT is an important factor influencing its activity. The dynamics of the changes in GGT activity depend on the genotypes of SNPrs5751901 and rs2236626, and it can reflect a different vulnerability of organism to oxidative stress. However, tobacco smoke exposure is an additional factor elevating the risk of AP occurrence by three times, especially in the individuals with the TC genotypes of SNP rs5751901. Exposure to tobacco smoke xenobiotics in the AP patients with the CC genotypes for both tested SNPs contributes to GGT loosening in the cell membrane, and molecule release with changed activity.

## Conclusions

- Single-nucleotide polymorphisms in the GGT1 gene encoding a large subunit (SNPs rs5751901 and rs2236626) causes changes in the activity of this enzyme.
- Exposure to tobacco smoke xenobiotics caused gradual increase in blood GGT activity in the course of AP in the patients with the TC and CC genotypes for SNP rs5751901 and rs2236626. Increased GGT activity in this group of AP patients contributes to excessive GSH use up during hospitalization of smokers as a result of neutralization of oxidative stress.
- The occurrence of the TC and CC genotypes for SNPs rs5751901 and rs2236626 predispose to differential GSH concentration depending on exposure to tobacco smoke. Smoke xenobiotics caused an increase in GSH reserves as a result of adaptive response to oxidative stress induced simultaneously by smoking and AP.
- OD analysis indicates more than 3-fold increase in the risk of acute pancreatitis occurrence in smoking individuals with the TC genotypes for SNP rs5751901.

## Supplementary Material

Supplementary figures and tables.

## Figures and Tables

**Table 1:** Clinical characteristics of the participants in the study.

Parameters	Healthy subjects	
	Non-smokers Mean ± SD	Smokers Mean ± SD
Age [years]	45,7 ± 8,7	46,1 ± 8,4
Gender [% female/% male]	85%/15%	48%/52%
BMI [kg/m^2^]	22,7 ± 1,6	23,0 ± 2,1
hsCRP [mg/l]	0,6 ± 0,2	0,4 ± 0,1
Cotinine [ng/ml]	3,3 ± 2,9	84,9 ± 5,0 ^1)^
**Parameters**	**Patients with AP**	
	**Non-smokers** **Mean ± SD**	**Smokers** **Mean ± SD**
Age [years]	52,2 ± 23,7	45,1 ± 12,3
Gender [% female/% male]	40%/60%	30%/70%
BMI [kg/m^2^]	28,2 ± 5,0	23,4 ± 4,5
The number of AP attacks in the past	0-3	0-10
Ranson Criteria [score]	2,5 ± 0,9	2,6 ± 0,6
hsCRP [mg/l]	163,2 ± 58,4	161,6 ± 63,2
Leukocytes [10^9^/l]	10,2 ± 5,6	11,2 ± 4,8
Erythrocytes [10^12^/l]	4,0 ± 0,7	4,1 ± 1,0
Hemoglobin [g/dl]	11,5 ± 1,5	12,2 ± 2,5
Hematocrit [%]	34,7 ± 4,0	36,4 ± 6,6
Bilirubin (total) [mg/dl]	1,1 ± 0,5	0,9 ± 0,6
ALAT [U/l]	37,4 ± 37,6	20,0 ± 14,5
ASPAT [U/l]	37,6 ± 28,9	27,7 ± 12,8
Alkaline phosphatase [U/l]	143,1 ± 76,2	143,0 ± 110,5
Lipase [U/l]	393,4 ± 488,9	518,3 ± 692,7
Glucose [mg/dl]	118,2 ± 28,3	102,7 ± 22,1
Urea [mg/dl]	29,3 ± 18,8	18,8 ± 1,0
Creatinine [mg/dl]	1,4 ± 1,8	1,0 ± 0,8
Cotinine [ng/ml]	7,6 ± 3,7	123,4 ± 59,9 ^1)^
Cd [µg/l]	1,3 ± 0,8	5,8 ± 2,8 ^1)^
AOPP [µmol/l]	48,2 ± 22,0	49,5 ± 17,2
oxLDL [U/l]	86,5 ± 39,6	95,2 ± 35,7
MDA [nmol/µl]	1,9 ± 0,3	2,4 ± 0,4 ^1)^

^1)^ p<0.05 compared to non-smokers.

**Table 2:** Characteristic of studied polymorphism.

Type of polymorphism	Chromosome/Chromosome position	Localization	Genotype	Primer's sequences
SNP, rs5751901	22/ 24596299	GGT1 (intron 1)	C/T	Forward: 5′ GGCAGAGTAAGGACCTGCCA 3′
				Reverse: 5′ GAATGCCATGTGAAGGCCAC 3′
SNP, rs2236626	22/ 24583479	GGT1 (5'region)	C/T	Forward: 5′ TCCTCACTCTCACTCCAGTGG 3′
				Reverse: 5′ cttcacgaccaccagcaagg 3′

**Table 3:** GGT1 genotypes for SNP rs2236626 and rs5751901 in patients with acute pancreatitis and healthy subjects.

	Patients with AP		Healthy subjects		Fischer's exact test
Genotypes	N=39	Frequency, %	N=51	Frequency, %	
**rs2236626**					
Homozygotes T/T	19	49 %	27	53 %	p=0,2186
Smokers/non-smokers	11/8	58% /42%	16/11	59% /41%	
Homozygotes C/C	6	15 %	8	16 %	p=0,1099
Smokers/non-smokers	1/5	17% /83%	3/5	37% /63%	
Heterozygotes C/T	14	36 %	16	31 %	p=0,0683
Smokers/non-smokers	11/3	78% /22%	8/8	50% /50%	
**rs5751901**					
Homozygotes T/T	15	38 %	24	47 %	p=0,2198
Smokers/non-smokers	8/7	53% /47%	14/10	58% /42%	
Homozygotes C/C	10	26 %	7	14 %	p=0,5468
Smokers/non-smokers	7/3	70% /30%	3/4	43% /57%	
Heterozygotes C/T	14	36 %	20	39 %	p=0,0447
Smokers/non-smokers	8/6	57% /43%	8/12	40% /60%	

**Table 4:** GSH concentration (mean ± SD) in the blood of AP patients divided in terms of SNPs rs5751901 and rs2236626.

GSH [µg/mL] concentration				
rs5751901				
Day of hospitalization		TT homozygotes	CC homozygotes	TC heterozygous
1^st^	Non-smokers	147,1 ± 68,7	79,6 ± 48,5	177,4 ± 128,7
	Smokers	185,6 ± 78,3	274,2 ± 51,4 1)	217,6 ± 114,9
3^rd^	Non-smokers	149,2 ± 85,7 2)	55,4 ± 9,0	79,6 ± 33,1
	Smokers	175,9 ± 53,7	233,7 ± 84,8 1)	164,1 ± 78,5 1)
7^th^	Non-smokers	158,7 ± 88,3 2)	40,4 ± 12,0	116,6 ± 34,2 2)
	Smokers	181,8 ± 47,7	161,3 ± 77,5 1), 3)	136,3 ± 65,9 3)
**rs2236626**				
1^st^	Non-smokers	214,9 ± 112,6 2), 4)	86,5 ± 20,2	89,0 ± 80,6
	Smokers	259,4 ± 141,2	204,6 ± 31,0 1)	197,6 ± 69,7 1)
3^rd^	Non-smokers	152,3 ± 97,6	106,8 ± 56,0	60,5 ± 2,3
	Smokers	193,2 ± 89,6	129,3 ± 22,5 3)	181,9 ± 62,1 1)
7^th^	Non-smokers	150,4 ± 109,3	67,4 ± 38,2	119,7 ± 4,4 5)
	Smokers	149,4 ± 80,7 3)	173,5 ± 23,5 1)	163,3 ± 45,6

1) p<0.05 compared to non-smokers. 2) p<0.05 compared to CC homozygotes. 3) p<0.05 compared to the 1^st^ day of hospitalization. 4) p<0.05 compared to TC heterozygous. 5) p<0.05 compared to the 3^rd^ day of hospitalization.

**Table 5:** Correlation coefficients for the group of smoking AP patients in terms of SNP rs5751901 and rs2236626.

	Correlated parameters	r	p
**SNP rs5751901**			
**TT genotype**	GGT activity - AOPP	0,8857	0,0456
	GGT concentration - AOPP	0,6163	0,0435
	GGT concentration - GSH	0,8763	0,0019
**TC genotype**	GGT activity - cotinine	0,5564	0,0072
	GGT activity - Cd	0,6200	0,0027
	GGT activity - Ranson score	0,7680	0,0438
	GGT activity - oxLDL	0,9671	0,0329
	GGT activity - MDA	0,7759	0,0403
	GGT concentration - GSH	0,6591	0,0382
**CC genotype**	GGT activity - hsCRP	0,6845	0,0290
	GGT activity - Cd	0,8386	0,0002
	GGT concentration - Cd	0,7784	0,0229
	GGT concentration - MDA	0,8847	0,0015
**SNP rs2236626**			
**TT genotype**	GGT activity - Cd	0,9786	0,0214
	GGT activity - hsCRP	0,6652	0,0131
	GGT activity - Ranson score	0,9931	0,0069
	GGT concentration - GSH	0,5174	0,0482
	GGT concentration - MDA	0,6359	0,0108
**TC genotype**	GGT activity - Cd	0,9992	0,0214
	GGT activity - hsCRP	0,9934	0,0068
	GGT activity - AP attacks	0,9994	0,0006
	GGT concentration - cotinine	0,6684	0,0490

## Abbreviations

AP: acute pancreatitis
GGT: γ-glutamyltransferase
GSH: glutathione
SNP: single-nucleotide polymorphism
