# Supplementary material for: Increased risk of acute pancreatitis occurrence in smokers with rs5751901 polymorphisms in GGT1 gene
Source: Int J Med Sci. 2020 Jan 14;17(2):242–54. doi: 10.7150/ijms.38657 (PMC6990886; doi:10.7150/ijms.38657)
Supplement: Supplementary file 1 — Supplementary figures and tables. [file ijmsv17p0242s1.pdf]

SUPPLEMENTARY MATERIAL

Genotypes and GGT activity in the blood of AP patients and healthy subjects

It was shown the highest GGT activity in the 1st day of hospitalization in the blood of AP patients with TT and TC genotypes for SNP rs5751901 (Figure 1). It was statistically significant compared to AP patients with CC genotypes for this SNP (p=0,0381 and 0,0315, respectively). In AP patients with TT genotypes for SNP rs5751901, GGT activity remained at the highest level in the 3rd day of hospitalization compared to individuals with CC (p=0,0358) and TC genotypes (p=0,0323) for this SNP. However, in the blood of AP patients with TT genotypes, 5-fold decrease in the activity of this enzyme in 7th day of hospitalization was observed, when compared to the 1st and the 3rd day (p=0,0432 and p=0,0404, respectively). A gradual decrease in GGT activity during hospitalization was also shown in the blood of AP patients with TC genotypes for SNP rs5751901 (p=0,0457 and p=0,0333 for comparison of the 1st day with the 3rd and 7th day, respectively) (Figure 1). In the case healthy subjects, it was observed no changes in GGT activity between individuals with each genotypes for SNP rs5751901 (Figure 1).

Figure 1. GGT activity and concentration in the blood of healthy subjects and the patients with AP in terms of SNP rs5751901.

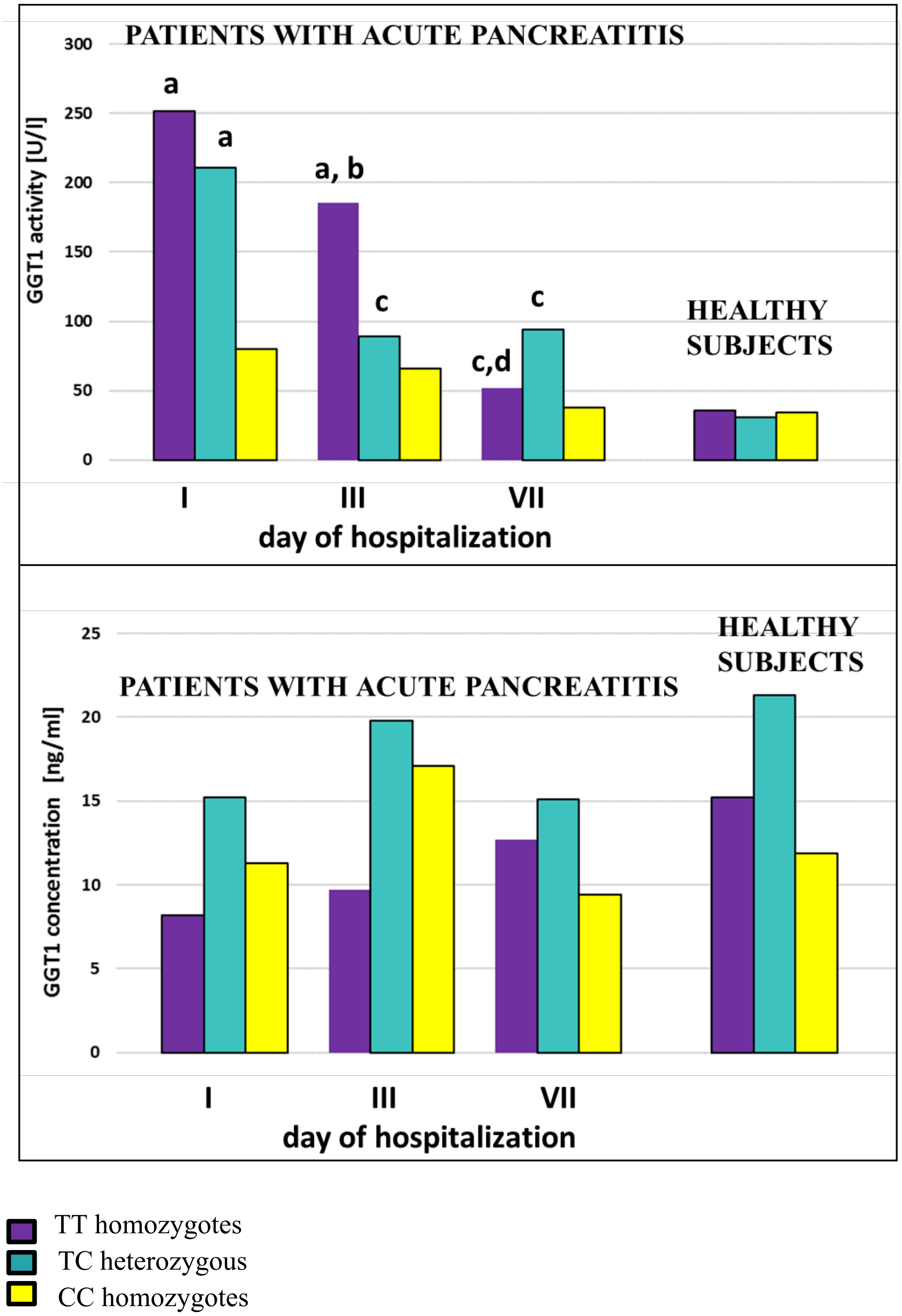

a – $p<0.005$ compared to CC homozygotes
b – $p<0.005$ compared to TC heterozygous
c – $p<0.005$ compared to the 1st day of hospitalization
d – $p<0.005$ compared to the 3rd day of hospitalization

In the blood of AP patients with CC genotypes for SNP rs2236626, the highest GGT activity was shown, what was statistically significant compared to individuals with TT genotypes (p=0,0153) (Figure 2). However, in the blood of these patients, a gradual decreasing GGT activity during hospitalization was shown. The activity of this enzyme was 2-fold lower in the 3rd day compared to the admission (p=0,0285) to be normalized in the 7th day (p=0,0040 compared to the 1st day). However, in the case healthy subjects, it was observed no changes in GGT activity between individuals with each genotypes for SNP rs2236626 (Figure 2).

Figure 2. GGT activity and concentration in the blood of healthy subjects and the patients with AP in terms of SNP rs2236626.

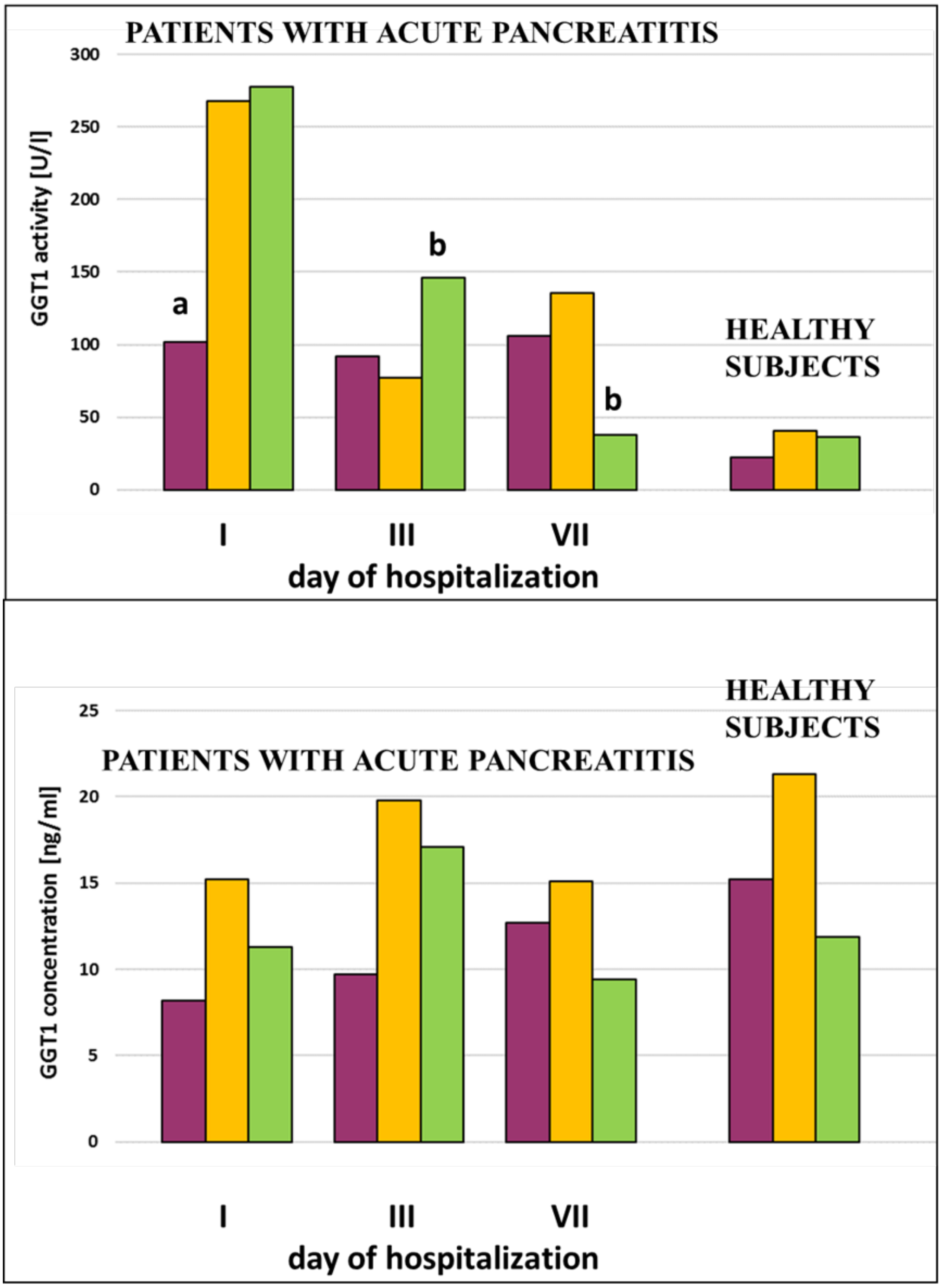

TT homozygotes
TC heterozygous
CC homozygotes

a – $p<0.005$ compared to CC homozygotes
b – $p<0.005$ compared to the 1st day of hospitalization
